# Alterations in Intestinal Microbiota Composition in Mice Treated With Vitamin D3 or Cathelicidin

**DOI:** 10.3389/fonc.2021.700038

**Published:** 2021-12-23

**Authors:** Yu Jiang, Yue Wan, Jing Li, Yueshui Zhao, Yongshun Ma, Jing Yu, Donghong Yuan, Shixin Xiang, Fukuan Du, Xu Wu, Mingxing Li, Yu Chen, Zhangang Xiao, Qinglian Wen, Wei Hu, Jing Shen

**Affiliations:** ^1^ Laboratory of Molecular Pharmacology, Department of Pharmacology, School of Pharmacy, Southwest Medical University, Luzhou, China; ^2^ South Sichuan Institute of Translational Medicine, Luzhou, China; ^3^ Laboratory of Personalised Cell Therapy & Cell Medicines, School of Pharmacy, Southwest Medical University, Luzhou, China; ^4^ Department of Oncology and Hematology, The Affiliated Traditional Chinese Medicine Hospital of Southwest Medical University, Luzhou, China; ^5^ Department of Gastroenterology, Shenzhen Hospital, Southern Medical University, Shenzhen, China

**Keywords:** gut microbiota, mCRAMP, vitamin D3, 16S rRNA sequencing, *L. lactis*

## Abstract

Gut microbiota is a complex aggregation of microbial organisms, which offers diverse protective benefits to the host. Dysbiosis of intestinal microbiota is frequently associated with many diseases. Vitamin D3 (VD), which was originally associated with bone health, also possesses antimicrobial activities and can act through antimicrobial peptide. Cathelicidin is a type of antimicrobial peptide in host to maintain the balance of gut microbiome. Our current study sought to evaluate the protective effect of VD and cathelicidin in mice intestines by administration of VD or mCRAMP-encoding *L. lactis*. We herein provided a comprehensive profile of the impact of VD and mCRAMP on gut microbiota using 16S rRNA sequencing, followed by bioinformatics and statistical analysis. Our results revealed an increased richness of bacterial community in mice intestines due to VD administration. Moreover, we showed a beneficial effect of VD and mCRAMP by enhancing the colonization of bacterial taxa that are associated with protective effects to the host but repressing the propagation of bacterial taxa that are associated with harmful effects to the host. Various metabolic pathways related to amino acid and lipid metabolism were affected in this process. We further established a bacterial panel as a reliable biomarker to evaluate the efficacy of remodeling the mice gut microbiota by VD and mCRAMP administration. The uncovered effects will deepen the comprehension about the antibacterial mechanisms of VD and mCRAMP and provide new insights for therapeutic implication of them.

## Introduction

The mammalian intestine harbors a complex and abundant aggregation of microbial organisms, including bacteria, viruses, fungi, and protozoa, which is collectively known as the gut microbiota (1–3). Depending on the advancement of culture-independent molecular methods, we now know that over 1,000 species of bacteria colonize inside human gastrointestinal tract, most of which belong to seven phyla: *Firmicutes*, *Bacteroidetes*, *Actinobacteria*, *Fusobacteria*, *Proterobacteria*, *Verrucomicrobia*, and *Cyanobacteria* (4, 5). Two phyla, *Firmicutes* and *Bacteroidetes*, account for >90% composition of the microbiome in healthy adult intestines (6, 7). It is established that these microorganisms in healthy individuals offer a diverse array of protective benefits to the host, which is correlated with nutrition, metabolism, and the immune system. Dysbiosis of intestinal microbiota is frequently associated with a plethora of diseases, including diabetes, obesity, liver and neuropsychiatric disorders, and inflammatory bowel diseases (IBD) (8, 9). Apart from these non-infectious health benefits, the gut serves as a portal of entry for communicating with the external environment. Extrinsic pathogenic microbes such as foodborne bacterial pathogens have to encounter and disrupt the balance of commensal microbiota, thus then successfully colonizing inside the mammalian intestine (10).

One of the strategies to defense against enteric infection is *via* the production of antimicrobial peptides (AMPs), which confers the antibacterial activities through a variety of mechanisms, including disruption of bacterial membranes and sequestration of necessary nutrients (11). AMPs are small molecules with a broad spectrum of antimicrobial activities against bacteria, fungi, and virus, serving as an ancient “porter” of the innate immune system (12). Cathelicidins, including mouse cathelicidin-related antimicrobial peptide (mCRAMP) and its human homologue LL-37, are a family of AMPs produced by animals and human, which acts as the first line of defense against pathogen infection (13, 14). To date, cathelicidin has been identified to be widely expressed by various types of cells, including monocyte/macrophage, mast cell, natural killer cell, and epithelial cell. In mammalian intestine, cathelicidin is constitutively secreted by enterocytes and Paneth cells, which are specialized epithelial cells localized in the small intestinal crypts (15). Abundant evidences have proved a broad antimicrobial activity of cathelicidin against bacteria, viruses, fungi, and parasites (16). Low concentrations of cathelicidin showed a fierce inhibition on the growth of multiple strains such as *Escherichia coli* through preventing biofilm formation (17). Similarly, a recent study reported an anti-hepatitis C virus (HCV) propagation effect of LL-37 in HuH-7 cell culture system (18). Moreover, defects in cathelicidin production are constantly correlated with susceptible infection with enteric pathogens such as *Listeria monocytogenes* (19, 20). Patients with ulcerative colitis (UC) or Crohn’s disease exhibited insufficient LL-37 secretion in the inflamed mucosa (21, 22), implying that restoration of cathelicidin production may contribute to the rebalance of gut microbiota in human intestine. We previously bioengineered a mCRAMP-secreting strain by using *Lactococcus lactis*, showing that replenishment with exogenous mCRAMP remarkedly restricted the colocalization of *Helicobacter pylori* in mice stomach as well as preventing the associated inflammatory responses (23, 24).

Vitamin D3 (VD), a hormone that primarily participated in maintenance of mineral homeostasis, has been revealed to promote a variety of extra-skeletal responses that may lead to a profound impact on human physiology (25). Prominent amongst VD-induced antimicrobial activities have been reported for different kinds of cell types and a range of pathogens (26). In particular, its anti-*Mycobacterium tuberculosis* (*M. tb*) activity has been reported for decades, which is closely related with the induction of cathelicidin (27). In the current study, we sought to explore the protective effect of VD and cathelicidin in mice intestines by administration of VD or mCRAMP-encoding *L. lactis*. Our results showed a profound impact by supplement with VD on the abundance and composition of mice gut microbiota, as evidenced by the increased alpha diversity (Represented by the Chao1 index). Moreover, reconstructed microbial composition with a beneficial effect was found for both VD or mCRAMP-encoding *L. lactis* administration by enhancing the colonization of bacterial taxa that are associated with beneficial effects to the host but repressing the propagation of bacterial taxa that are associated with harmful effects to the host.

## Materials and Methods

### Bacterial Strains

*Lactococcus lactis* (*L. lactis*) strains NZ3900 expressing the vector (Control) or mCRAMP were kind gifts from Prof. CHO Chin Hin (The Chinese University of Hong Kong, Hong Kong). *L. lactis* was maintained in M17 broth containing 50% (v/v) glycerol at −80°C and defrozen in M17 broth before each experiment. Induction of mCRAMP expression was performed as previously described (23, 24). Briefly, Control or mCRAMP-encoding *L. lactis* were incubated in M17 broth at 30°C without aeration overnight, and diluted in a fresh broth in 1:25 ratio, then incubated until A600 reached 0.4–0.5. Nisin (250 pg/ml) as an inducer was added to Control or mCRAMP-encoding *L. lactis* broth and further incubated for 3 h. Bacterial suspensions were harvested by centrifugation (8,000 rpm, 5 min) and resuspended with sterilized PBS to the desired concentration: 1 × 10^11^ colony-forming units (cfu) per ml.

### Animals and Treatments

**Figure 1:**
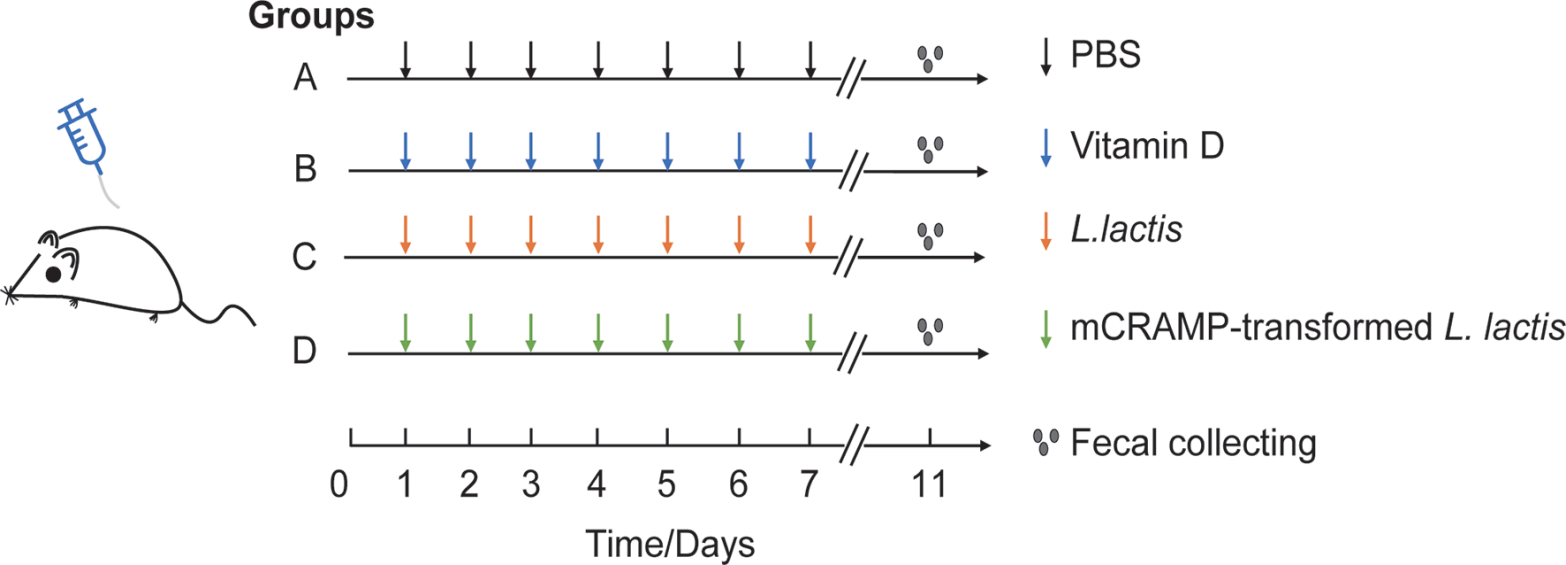
Experimental design for mice model. Mice in group A, B, C, and D were orally given PBS (100 μl), VD (25 μg/kg), *L. lactis* (10^10^ cfu/mouse), or mCRAMP-encoding *L. lactis* (10^10^ cfu/mouse), respectively, every day from day 1 to day 10. Stool samples were collected on day 11 morning for further analysis.

Male Balb/c mice (5–7 weeks old) were purchased from Specific-pathogen-Free (SPF) Biotechnology Co., Ltd (Beijing), and housed in the animal laboratory center of Southwest Medical University under SPF condition. All animals were acclimatized to the environmental conditions for 7 days before allowed free access to standard autoclaved lab diet and tap water under 22 ± 2°C room temperature with a 12 h light/dark cycle. Mice were randomly separated into 4 groups: PBS group (12 mice), VD-treated group (10 mice), *L. lactis*-treated group (N0I) (10 mice), and mCRAMP-transformed *L. lactis*-treated group (N4I) (10 mice). From day 1 to day 10, mice were given daily of 0.1 ml autoclaved PBS, VD (25 μg/kg in 0.1ml PBS), 10^10^ cfu control *L. lactis*, or 10^10^ cfu mCRAMP-transformed *L. lactis* in 0.1 ml PBS by intragastrical administration, respectively (**Figure 1**). All animal experiments were approved by the Committee on Use and Care of Animals of Southwest Medical University. And all experimental protocols were performed in accordance with the experimental guidelines and regulations of Southwest Medical University.

### Sample Collection and DNA Extraction

Mice fecal samples were collected at the end of experiment on Day 11. All samples were quickly frozen with liquid nitrogen and immediately stored at −80°C until DNA extraction.

Microbial DNA was extracted using E.Z.N.A. Soil DNA Kit (Omega Bio-tek, Norcross, GA, USA) according to the manufacturer’s instruction. The quantification of DNA was detected by NanoDrop2000 (Thermo Electron). DNA integrity and size were evaluated by 1.0% agarose gel electrophoresis.

### PCR and 16s rRNA Sequencing

The primer pairs 338F (5’-ACT CCT ACG GGA GGC AGC AG-3’) and 806R (5’- GGA CTA CHV GGG TWT CTA AT-3’) were used to amplify the bacterial V3-V4 hypervariable regions of 16s rRNA gene by thermocycle PCR system (GeneAmp 9700, ABI, USA). The PCR reactions were carried out as Yin J et al. (28) described. The PCR products were collected and purified, and the amplicons were further sequenced on an Illumina MiSeq platform (Illumina, San Diego, USA) according to the standard protocols by Majorbio Bio-Pharm Technology Co. Ltd. (Shanghai, China) (28).

### Microbial Analysis

Raw fastq files were quality-filtered by Trimmomatic and merged by FLASH, then normalized the number of sequences on the shortest sample sequence level to control for coverage differences between the samples or other conditional sequencing events and to ensure even sampling of the reads. The sequences were classified on 97% similar levels of OTU representative sequences using UPARSE (version 7.1 http://drive5.com/uparse/), and the chimeric sequences were identified and removed using UCHIME. The taxonomy of each 16S rRNA gene sequence was analyzed by RDP classifier Bayesian algorithm reference to silva database (Release132 http://www.arb-silva.de). All OTUs were classified from phylum down to the species level. The 16s rRNA raw date are accessible in NCBI with the accession number of SRP275495.

Rarefaction curve and Alpha diversity, including Chao1 index and Shannon index, were calculated using mothur version v.1.30.1. Distance heatmap and principal coordinates analysis (PCoA) were analyzed in R tools using Bray-Curtis dissimilarity matrices. LEfSe cladograms and Linear Discriminant Analysis (LDA) were performed using LEfSe software (http://huttenhower.sph.harvard.edu/galaxy/root?tool_id=lefse_upload) to explore different bacteria from phyla to genus level among the treatments. R package was used to calculate the Area Under the ROC Curve (AUC). Based on 16s rRNA gene sequence data and quantification, KEGG pathway and COG function were predicted using Phylogenetic Investigation of Communities by Reconstruction of Unobserved States (PICRUSt2), referencing to Kyoto Encyclopedia of Genes and Genomes (KEGG) database, MetaCyc Metabolic Pathway database, and Evolutionary Genealogy of Genes: Non-supervised Orthologous Groups (EggNOG) database.

### Statistical Analysis

Data were analyzed on the free online platform of Majorbio Cloud Platform. Welch’s t-test was used to evaluate statistically significant differences of alpha diversity index; KEGG pathway and COG function between groups and Wilcoxon rank-sum test were used to identify statistically significant differences of microbial abundance between groups. *P-*value <0.05 was considered as a statistical difference. Differences of microbial community structure were evaluated by permutational multivariate analysis of variance (PERMSNOVA) (also named Adonis) analysis at 999 permutations. ANOSIM analysis was used to test whether the differences between groups are significantly greater than the differences within groups through vegan package of R tools by Bray-Curtis algorithm. *P-*value <0.05 was noted to a significant difference.

## Results

### OTU Generation

To characterize the gut microbiome community dynamics in mice intestines, we collected fecal specimens from four groups of experimental mice designated as PBS group (PBS, N=12), VD group (VD, N=10), *L. lactis* group (N0I, N=10), and mCRAMP-transformed *L. lactis* group (N4I, N=10). Bacterial DNA were extracted from fecal samples and then subjected to 16S rRNA V3V4 amplicon sequencing on an Illumina MiSeq system, followed by bioinformatics and statistical analyses. Resultingly, we obtained 2,380,966 high-quality 16S rRNA merged reads and 1,013,239,002 bases (bp) from 42 samples in total. The average length was 425.558 bp. More information about the sequence of each mouse are listed in **Supplementary Table S1**. After normalized to the minimum sample sequence number, these reads were then clustered into 667 OTUs (Corresponding to 12 phylum, 18 classes, 32 orders, 59 families, 132 genus, and 211 species), and summarized in **Supplementary Table S2**, which will be used for the following analysis.

### Upregulated Richness and Diversity of Intestinal Microbiota Community in VD and mCRAMP-Encoding *L. lactis*–Administrated Mice

**Figure 2:**
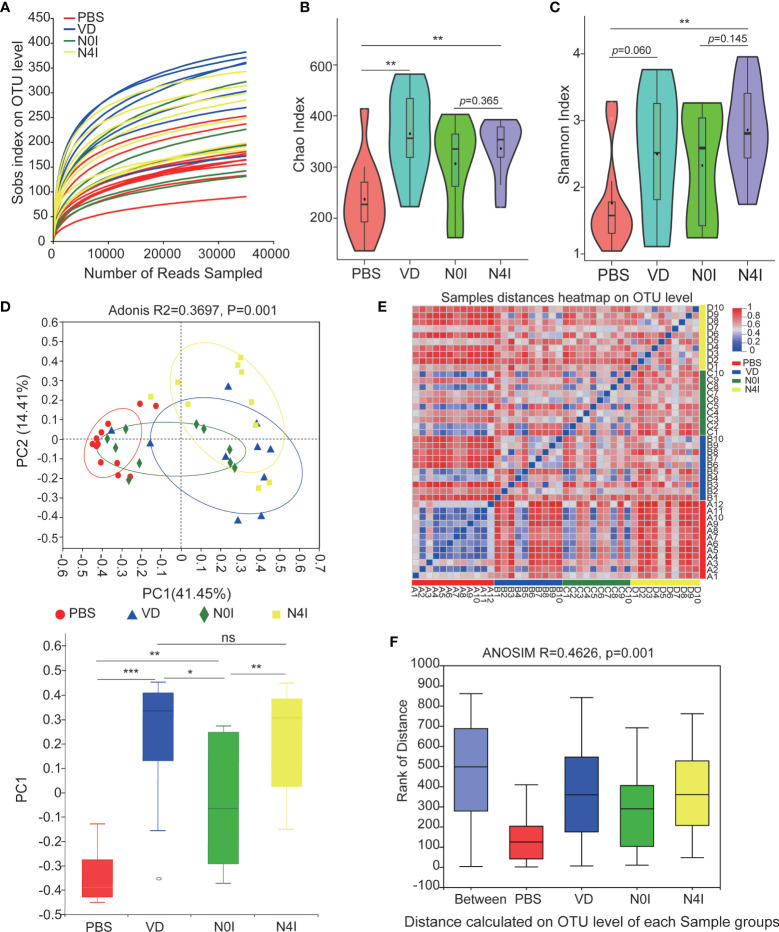
Diversity estimate calculations of bacterial OTUs between control, VD, and mCRAMP-encoding *L. lactis*–treated mice. **(A)** Rarefaction curve was generated to determine the sequencing depth per sample. **(B, C)** The Chao1 and Shannon indexes of control, VD, and mCRAMP-encoding *L. lactis*–treated mice were calculated to estimate bacterial richness and diversity. Horizontal lines represent mean value. ^**^ *p* < 0.01. *p* < 0.05 indicates statistical significance using a Welch’s t-test. **(D)** Principal component analysis (PCoA) were used to evaluate the bacterial community difference in each group by Bray-Curtis. PERMANOVA (Adonis) analyses were performed to evaluate the overall differences on OTU level between groups. The box plot showed the difference of microbial community on the first principal coordinate (PC1), and the statistical differences were determined with Mann-Whitney test (***p < 0.001, **p < 0.01, *p < 0.05). **(E)** Heatmap using the Bray-Curtis distances was analyzed to assess sample distances at genus level based on the OTU results. **(F)** Distance boxplot with ANOSIM analysis indicated a significant difference overall the groups of samples (p < 0.05). **(B, C)** Were generated using an online tool ImageGP (http://www.ehbio.com/ImageGP/index.php/Home/Index/index.html), and **(E)** was performed by R software using vegan package. ns, not significant.

The adequacy of sampling depth in each sample was estimated by rarefaction analysis (**Figure 2A**). Despite deep sequencing we did not identify all possible OTUs within a sample, as demonstrated by the rarefaction curves. Microbiota richness and diversity were then assessed by Chao1 (**Figure 2B**) and Shannon (**Figure 2C**) index. Mice administrated with VD showed a consistent elevation in microbiota richness as compared with the PBS group, which was evidenced by the increased Chao1 index (p < 0.01) (**Figure 2B**). Mice receiving mCRAMP-tranformed *L. lactis* also demonstrated elevated Chao1 index, which was not significantly different from mice receiving *L. lactis*, indicating this effect was possibly due to *L. lactis* but not mCRAMP. Shannon index also revealed an enhanced microbiota diversity after introducing mCRAMP-transformed *L. lactis* in mice intestines. There was a trend of difference between *L. lactis*, and mCRAMP-transformed *L. lactis* groups, but it didn’t reach statistical significance. No statistical significance was found between PBS and VD group (**Figure 2C**). Next, bacterial community difference in each group was evaluated using the scatter plot based on our principal coordinate analysis (PCoA), which shows significant distinction in mice with different treatments (Adonis, R^2 =^ 0.3697, p=0.001), and the bar plots represented significantly different distribution of different groups of the fecal samples on the first principal coordinate (PC1) (**Figure 2D**). Heatmap was also generated to visualize the difference of microbiota composition at genus level based on the OTU results. Consistently, we observed distinct clusters of bacterial community composition in VD and N4I groups as compared with control mice (**Figure 2E**). The analysis of similarities (ANOSIM) verified that the microbial composition was significantly different among groups (p = 0.001) (**Figure 2F**). Thus, our data collectively revealed a reconstruction effect of microbiota community after VD or mCRAMP-encoding *L. lactis* treatment.

### Reshaped Microbiota Community Composition at Phylum and Genus Level in VD and mCRAMP-Encoding *L. lactis*–Administrated Mice Intestines

**Figure 3:**
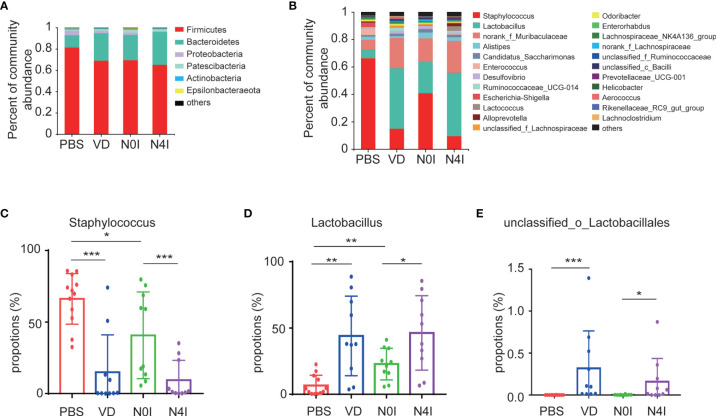
Relative abundance of different bacteria in control, VD, and mCRAMP-encoding *L. lactis*–treated mice. Bacteria taxonomic profiling at the phylum **(A)** and genus **(B)** levels of gut microbiota from control, VD, and mCRAMP-encoding *L. lactis*–treated mice were presented. **(C–E)** Bar graphs show the relative abundance of bacteria in control, VD, and mCRAMP-encoding *L. lactis*–treated mice. ^*^ *p* < 0.05; ^**^ *p* < 0.01; ^***^ *p* < 0.001. *p* < 0.05 indicates statistical significance using a nonparametric Mann-Whitney U test.

Taxonomic analysis based on the OTU results revealed a presence of six phyla, with Firmicutes and Bacteroidetes being the dominating phyla accounting for about 71.77 and 22.41% of the total reads, respectively. Other bacteria from *Proteobacteria*, *Patescibacteria*, *Actinobacteria*, and *Epsilonbacteraeota* taxa account for the rest of 6.7% in the PBS group (**Figure 3A**). Alterations in the relative abundance of Firmicutes and Bacteroidetes were observed in mice treated with VD or mCRAMP-encoding *L. lactis*. Compared to PBS or N0I group, the abundance of Firmicutes decreased 12.32% (p = 0.1765) and 4.31% (p = 0.5708), respectively, whereas Bacteroidetes amount increases 14.22% (p = 0.0927) and 7.08% (p = 0.4274) in mice treated with VD or mCRAMP-encoding *L. lactis* (**Figure 3A**).

At the genus level, OTUs were assigned to 132 individual genera, of which 23 were present in all specimens with a relative abundance of more than 0.5% in at least one sample. Seven genera dominated in control mice with a proportion of more than 91.35%, whereas the ranking of bacterial composition shifted after mice treated with VD or mCRAMP (**Figure 3B**). Interestingly, several pernicious bacteria, including *Staphylococcus*, *Escherichia-Shigella*, and *Anaerotruncus*, were revealed to be repressed by VD or mCRAMP-encoding *L. lactis* (**Figure 3C** and **Supplementary Table S3**) . On the contrary, beneficial bacteria such as *Lactobacillus* and *unclassified_o_Lacobacillales* showed an enhanced colonization in mice gut by VD and mCRAMP administration (**Figures 3D** **, E**). After further comparison, we found VD but not mCRAMP may enrich the abundance of *Aerococcus*, *Faecalibaculum*, and *UBA1819*, while mCRAMP could specifically inhibit *unclassified_K_norank_d_Bacteria* (**Supplementary Figure 1**). Amongst them, *Staphylococcus* is an established risk factor for gastrointestinal infection (29), while *Lactobacillus* is a kind of probiotic that has a beneficial impact on stress response and depressive disorder (30, 31). Although some changes of the bacteria were also found in *L. lactis*–administrated mice, the changes in mCRAMP-encoding *L. lactis* mice were statistically significant as compared to the *L. lactis*–administrated mice. We herein discovered a restrained colonization of harmful bacteria as well as prompted bacterial taxa that are associated with beneficial effects to the host propagation in mice intestines after VD and mCRAMP treatments, indicating a protective role of them in mice gut. Thus, our results demonstrated a reshaped bacterial community composition with a setting right impact by facilitating the beneficial bacteria but restraining the harmful bacterial colonization in mice intestines after being treated with VD or mCRAMP.

### Different Bacterial Diversity and Composition in Control, VD, and mCRAMP-Encoding *L. lactis*–Administered Mice

**Figure 4:**
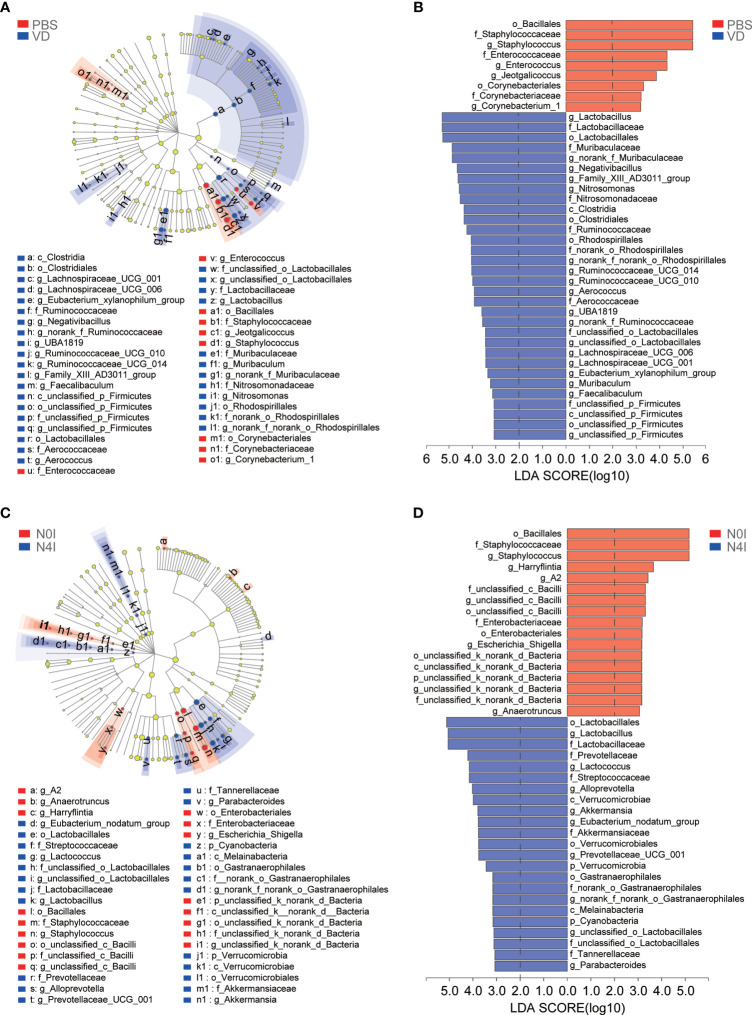
LEfSe analysis of gut microbiota taxa in control, VD, and mCRAMP-encoding *L. lactis*–treated mice. **(A, C)** LEfSe cladograms were built to show the differently abundant bacteria in each group. Red nodes represent the bacterial biomarkers with highest abundance in PBS **(A)** or N0I **(C)** groups, while blue nodes indicate the predominant bacterial biomarkers in VD **(A)** or N4I **(C)** groups. “p” referred to phylum, “c” referred to class, “o” referred to order, “f” referred to family, “g” referred to genus. **(B, D)** Bar plots show linear discriminant analysis (LDA) scores, which are interpreted as the degree of consistent difference in relative abundance of fecal bacterial communities across each group. Red bars represent the bacterial biomarkers with highest abundance in PBS **(B)** or N0I **(D)** groups, while blue bars indicate the predominant bacterial biomarkers in VD **(B)** or N4I **(D)** groups. LDA score greater than 2 was considered a difference in bacterial abundance among each group. The LEfSe analysis was analyzed by LEfSe software (http://huttenhower.sph.harvard.edu/galaxy/root?tool_id=lefse_upload).

Next, linear discriminant analysis effect size (LEfSe) approach was applied to identify the discriminant bacterial species in control and mCRAMP-administrated mice in order to assess the detailed discrepancy in bacterial diversity and composition of mice intestines. Firstly, we presented the cladogram to reveal differences in 41 taxa between PBS and VD groups (**Figure 4A**) and 40 taxa between N0I and N4I groups (**Figure 4C**). The plots from LEfSe analysis displaying LDA scores of microbial taxa were further generated. LDA scores greater than 2 were considered a difference in bacterial abundance across 4 treatment groups. Resultingly, our data revealed that 9 clades, including *o_Bacillales*, *f_Staphylococcaceae*, *g_Staphylococcus*, *f_Enterococcaceae*, *g_Enterococcus*, *g_Jeotgalicoccus*, *o_Corynebacteriales*, *f_Corynebacteriaceae*, and *g_Corynebacterium_1*, were associated with control subjects (**Figure 4B**). On the contrary, 32 taxa were showed to be the most significantly abundant taxa, of which *g_Lactobacillus*, *f_Lactobacillaceae*, *o_Lactobacilales*, *f_Muribaculacease*, and *g_norank_f_Muribaculaceae* were the five most dominant in VD-treated mice (**Figure 4B**). Interestingly, we observed similarly abundant bacterial taxa in N0I and control groups, and also similarly abundant bacterial taxa in N4I and VD-treated mice intestines. Taxa, including *o_Bacillales*, *f_Staphylococcaceae*, and *g_Staphylococcus*, were also showed to be abundant in N0I group, whereas accumulated *o_Lactobacillus*, *g_Lactobacillaceae*, *f_Lactobacilales* were consistently observed in mCRAMP-encoding *L. lactis*–administrated mice (**Figure 4D**). A more detailed comparison between different groups is shown in **Supplementary Figure 2**. Thus, our findings collectively indicated that VD and mCRAMP-encoding *L. lactis* parallelly reshaped the community composition of gut microbiome by facilitating the colonization of bacterial taxa that are associated with beneficial effects to the host as well as restricting the propagation of bacterial taxa that are associated with risky effects to the host in mice intestines.

**Figure 5:**
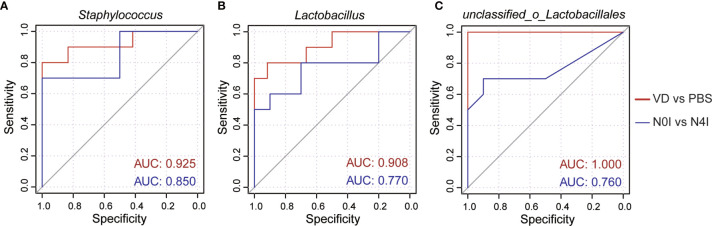
The area under curves of potential bacterial biomarkers in discriminating mCRAMP-treated mice from control subjects. **(A–C)** ROC curves of three selected bacterial marker candidates, including *Staphylococcus* **(A)**, *Lactobacillus* **(B)**, and *unclassified_o_Lacobacillales* **(C)**, were calculated to determine the efficacy of reshaped gut microbiota composition in mCRAMP-treated mice. AUC values close to 1 indicate that a high true positive rate was achieved with low false positive rate (ideal performance), while AUC values close to 0.5 indicate random performance.

To further evaluate the efficacy of remodeling mice gut microbiota *via* introducing VD and mCRAMP, we selected *Staphylococcus*, *Lactobacillus*, and *unclassified_o_Lacobacillales* as the bacterial biomarker panel to generate ROC curves, as these three bacteria were observed the most significantly altered species in mice intestines due to VD and mCRAMP treatment. Resultingly, the area under the curve (AUC) values ranging from 0.760 to 1 were revealed after treatment with VD or mCRAMP-encoding *L. lactis* (**Figure 5**), showing them reliable biomarkers to discriminate the control and VD or mCRAMP-treated mice.

### Predicated Gut Microbiota Function Using Picrust

**Figure 6:**
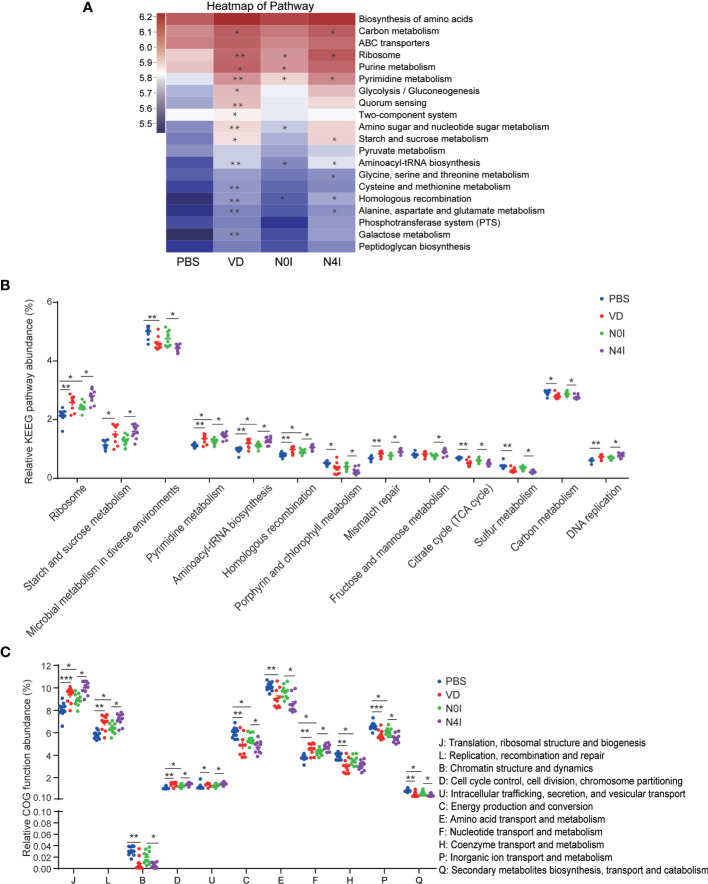
Differentially enriched functions of gut microbiota among control, VD, and mCRAMP-treated mice by PICRUSt analysis. The functions were predicated by KEGG **(A, B)** and COG **(C)** database in control, VD, and mCRAMP-treated mice. **(A)** Heatmap showing the top 20 pathways of the abundance, which was colored by logarithmic value, and the significant difference between the groups (VD *vs* PBS, N0I *vs* PBS, N4I *vs* N0I) were marked. **(B)** Pathways with over 0.1% changes compared to control group were shown. **(C)** The significantly different COG functions. ^*^ *p* < 0.05; ^**^ *p* < 0.01. ^***^ *p* < 0.001 indicates statistical significance using Welch’s t-test and adjusted false discovery rate. The heatmap was analyzed using PICRUSt package by R software, and **(B, C)** were generated by Graphpad Prism 7 (GraphPad Software Inc, LaJolla, CA, USA).

To gain insight into the molecular functions of bacteria, we adopted the Phylogenetic Investigation of Communities by Reconstruction of Unobserved States (PICRUSt) to infer the functional gene composition of gut microbiota by VD and mCRAMP treatment (32, 33). By variance analysis of Kyoto Encyclopedia of Genes and Genomes (KEGG) metabolic pathways, we observed that lots of metabolic pathways were changed by VD and mCRAMP. The top 20 abundant metabolic pathways are shown in the heatmap (**Figure 6A**). At least 7 metabolic pathways, including ribosome, starch, and sucrose metabolism, pyrimidine metabolism, mismatch repair, carbon metabolism, and DNA replication, were significantly increased by VD and mCRAMP treatment, whereas microbial metabolism in diverse environments, porphyrin and chorophyll metabolism, citrate cycle (TCA cycle), and sulfur metabolism were decreased significantly (**Figure 6B**). Additionally, the Clusters of Orthologous Groups (COG) database was used to predict the abundance of bacterial functions, revealing an enrichment of 5 functional categories including “translation, ribosomal structure and biogenesis”, “replication, recombination and repair”, “cell cycle control, cell division, chromosome partitioning”, “intracellular trafficking, secretion, and vesicular transport”, and “nucleotide transport and metabolism” in VD and mCRAMP-administrated mice intestines, and a decline of 4 functional categories, including energy production and conversion, amino acid transport and metabolism, inorganic ion transport and metabolism, and secondary metabolites biosynthesis, transport, and catabolism (**Figure 6C**).

## Discussion

According to the estimates from the literature, there are 3.8×10^13^ bacterial cells in the human body, which is the same magnitude to the number of human cells (34). Accumulating evidences have established an essential role of the structures and components of gut microbiota on the balance between health and disease and may be considered as an additional organ in human. Interactions with immune, central nervous, and enteric nervous system may provide the opportunity for certain probiotic species and strains to modulate the gut microbial composition, which is beneficial to the host (35). Disrupted gut microbiome environment and composition led to a diversity of physical dysfunction, including weight loss, diabetes, neurological diseases (e.g., schizophrenia), intestinal diseases, heart failure, hepatocellular carcinoma, and even cancer (36).

In the current study, we provided a comprehensive profile with regard to the reconstruction impact of VD and antimicrobial peptide cathelicidin on gut microbiota by administration of VD or mCRAMP-encoding *L. lactis* in mice intestines. By examining the mice gut microbiome with 16S rRNA sequencing, we uncovered that, at phylum level, *Firmicutes* and *Bacteroidetes* comprised the most two abundant microbial species in control mice gut, which are also the most predominant phyla in human intestinal microbiota (37). A previous evidence indicated that an elevated bacterial composition of *Firmicutes* was correlated with obesity (38). Conversely, a decreased *Firmicutes* proportion has been directly related to weight loss. *Bacteroidetes* as another dominant species mostly inhabits the distal gut, providing the host with energy through polysaccharides fermentation with thousands of enzyme combinations (39, 40). Overnutrition diet was proved to reduce the abundance of *Bacteroidetes*, which was positively correlated with obesity and non-alcoholic fatty liver diseases (NAFLD) (41, 42). Our data revealed a restricted colonization of *Firmicutes* but an enhancement on *Bacteroidetes* growth owing to extra supplement of mCRAMP in mice gut, suggesting a modulatory function of such peptide on host metabolic processes. It was further substantiated by KEGG and COG analysis, showing that mCRAMP administration mainly interferes with various metabolic pathways related to amino acid and lipid metabolism. When specific to the genus level, *Staphylococcus* is a well-known pathogenic bacterium recently identified to colonize inside the intestinal tracts of hospitalized patients, which may be an important reason for various gastrointestinal disorders such as diarrhea (29, 43). Similarly, *Escherichia-Shigella* is a common pathogen to trigger inflammation of the colon mucosa, leading to symptoms such as diarrhea, abdominal pain, and mucosanguineous stool (44). In the present study, we reported a repressive effect of mCRAMP in the colonization of *Staphylococcus* and *Escherichia-Shigella* in mice intestines (**Supplementary Table S3**). In contrast, *Lactobacillus* and *unclassified_o_Lactobacillales* are kinds of beneficial bacteria considered as symbiotic microorganisms to positively affect the health of host through secreting certain metabolic products (e.g., lactic acid, bacteriocins, and phenyllactic acid) or to maintain intestinal integrity (45). Our sequencing data showed a facilitated effect of VD and mCRAMP on the colonization of such beneficial bacteria in mice gut. Therefore, the current study uncovered a beneficial effect of VD and mCRAMP by prompting colonization of bacterial taxa that are beneficial to the host but repressing bacterial taxa that are associated with harmful effects to the host in mice intestines.

In this research, we further applied LEfSe approach to discriminate taxa that are differentially enriched in either control or mCRAMP-administrated mice. Multiple taxa were identified to be altered owing to administration of VD and mCRAMP. For instance, *o_Bacillales*, *f_Staphylococcaceae* and *g_Staphylococcus* were the most dominant species in control mice. However, consistent with upregulated Chao1 index, we revealed an increased bacterial diversity and richness, usually representing a “healthier” intestinal microbiome, in VD-treated mice intestines. Consequently, a high ranking of various beneficial bacteria, including *g_Lactobacillus*, *f_Lactobacillaceae*, and *o_Lactobacillales*, was observed due to VD administration. *Lactobacillus* is the largest genus within the group of lactic acid bacteria (30, 46). As the normal flora that usually inhabits in human gastrointestinal and urinary system, *Lactobacillus* is a type of “friendly” bacteria that produces lactic acid (leading to a low pH) and competitively inhibits pathogenic organisms without causing disease, which has been used in alternative medicine as a likely effective aid in preventing diarrhea and Irritable Bowel Syndrome (IBS) (47). In this study, we proved an accelerated effect of VD treatment to favor the colonization of such bacteria in mice. Importantly, our data revealed a parallel impact by using mCRAMP-encoding *L. lactis* treatment, that is, repressed colonization of harmful bacterial as well as enhanced growth of beneficial bacteria.

The 16S rRNA sequencing analysis has been extensively used in the classification and identification of bacteria and archaea, which is a particularly useful tool to understand the structure, dynamics, and relationship of microbial community (48). However, as compared with other emerging massively parallel sequencing technologies such as Whole Genome Shotgun (WGS) metagenomics sequencing, 16S rRNA-based techniques are known to have several limitations, including low resolution, short read lengths obtained, sequencing errors, and difficulties in assessing OTUs (49). In the current research, 16S rRNA sequencing technique was adopted to determine the alteration of mice intestinal microbiota community in response to VD and mCRAMP, which is a rapid and accurate identification method for bacterial adscription. Nevertheless, due to the limitations of such technology, we can only provide resolution till genus level and present nucleotide variations in rRNA operons in a single genome. Thus, metagenome approaches such as shotgun will still be needed to offer a higher resolution of the microbiota composition at species level in the future. Our PICRUSt functional analysis should also be verified by metagenomic shotgun sequencing by then. Moreover, our present study used *Lactococcus lactis* as the carrier to specifically secret mCRAMP in mice gut, which has been proved by previous evidences showing that *Lactococcus* was an appropriate tool to produce certain ingredients in gastrointestinal tract, thus to treat diseases such as acute colitis (50). However, *Lactococcus lactis* administration alone should have some impact on the structure and composition of mice gut microbiota, which should be taken into account when using this microorganism as drug delivery carrier in the future. In addition, we only analyzed fecal samples in this study. However, the induction of VD and mCRAMP may be enhanced in the bacteria that is close to the epithelium, which warrants further study.

In summary, we herein offered a comprehensive description regarding the reshaped structure and composition of mice gut microbiota due to administration of VD or mCRAMP-encoding *L. lactis*. Our presented findings uncovered a setting right effect of VD and mCRAMP by favoring the colonization of beneficial bacteria but inhibiting the risky bacterial growth in mice intestines, indicating an advantage of such supplementation in maintaining the “healthy” microbiota community in host gut. The uncovered effects will deepen the comprehension about the antibacterial mechanisms of VD and mCRAMP as well as providing new insights for therapeutic implication of this peptide in the treatment of human disease in the future.

## Data Availability Statement

The datasets presented in this study can be found in online repositories. The names of the repository/repositories and accession number(s) can be found below: https://www.ncbi.nlm.nih.gov/, SRP275495.

## Ethics Statement

The animal study was reviewed and approved by Committee on Use and Care of Animals of Southwest Medical University.

## Author Contributions

YJ, YW, and JL performed experiments. All authors contributed intellectually to the project through discussion and critically reviewed the manuscript. JS designed the study. JS, WH, and QW managed the project. All authors contributed to the article and approved the submitted version.

## Funding

This work was supported by National Natural Science Foundation of China (Nos. 81974070, 81972643, and 81800503), National Key R&D Project of China (No. 2018YFC0115301), Guangdong Basic and Applied Basic Research Foundation (2020A1515011063), Sichuan Science and Technology Project (2018JY0079 and 2021YJ0201).

## Conflict of Interest

The authors declare that the research was conducted in the absence of any commercial or financial relationships that could be construed as a potential conflict of interest.

## Publisher’s Note

All claims expressed in this article are solely those of the authors and do not necessarily represent those of their affiliated organizations, or those of the publisher, the editors and the reviewers. Any product that may be evaluated in this article, or claim that may be made by its manufacturer, is not guaranteed or endorsed by the publisher.
