# Supplementary figures and images for: Alterations in Intestinal Microbiota Composition in Mice Treated With Vitamin D3 or Cathelicidin
Source: Front Oncol. 2021 Dec 23;11:700038. doi: 10.3389/fonc.2021.700038 (PMC8732771; doi:10.3389/fonc.2021.700038)

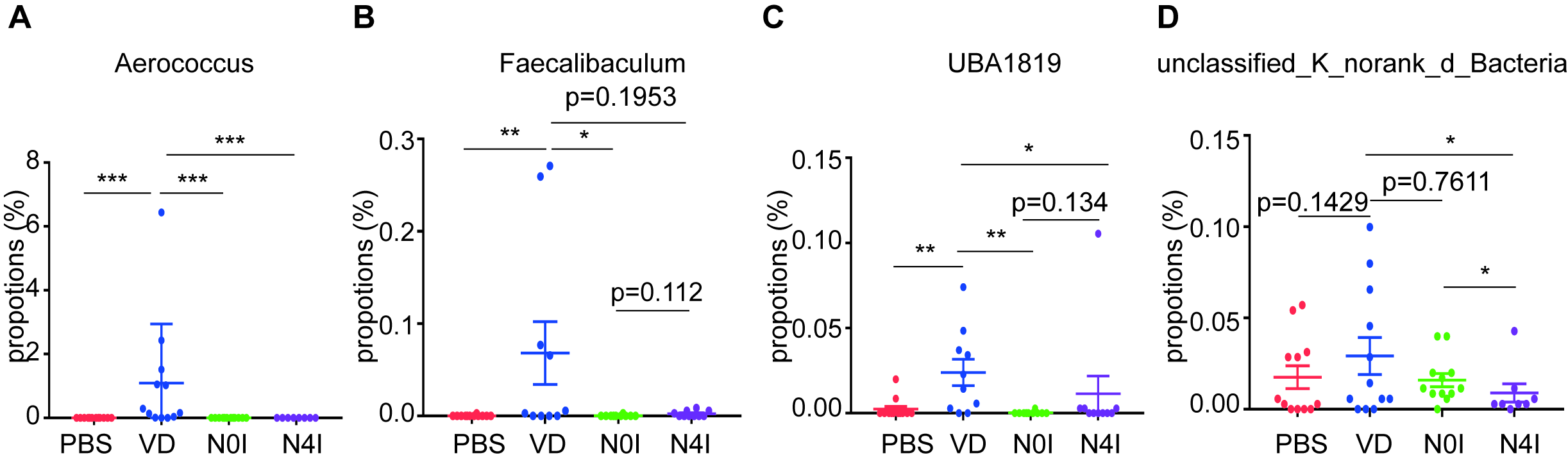

Supplement: Supplementary file 1 [file Image_1.tif]

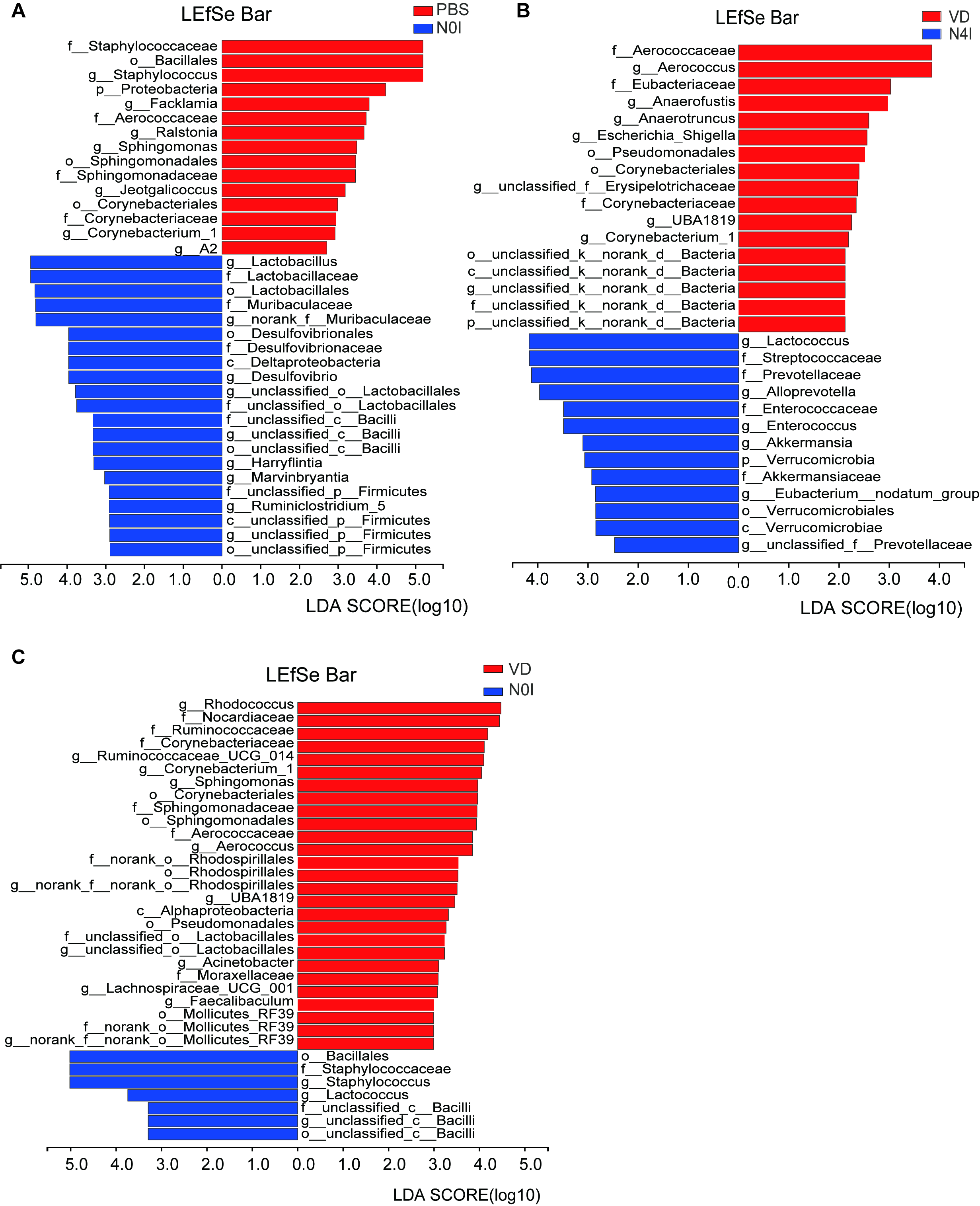

Supplement: Supplementary file 2 [file Image_2.tif]
